# The transition from preclinical to clinical obesity: the importance of a borderline stage

**DOI:** 10.1042/CS20256728

**Published:** 2026-01-07

**Authors:** Jesus E. Maldonado-Arvizu, Paula Vanessa Rios-Verdugo, José Fernando Díaz-Villanueva, Brenda Chimal-Vega, José L. Vique-Sánchez, Victor García-González

**Affiliations:** 1 Departamento de Bioquímica, Facultad de Medicina Mexicali, Universidad Autónoma de Baja California, Mexicali, Baja California, México; 2 Laboratorio Multidisciplinario de Estudios Metabólicos y Cáncer, Facultad de Medicina Mexicali, Mexicali, Baja California, México; 3 Centro de Investigación e Innovación en Salud (CIIS), Universidad Autónoma de Baja California, Mexicali, 21000, México; 4 Centro de Ciencias de la Salud Mexicali, Universidad Autónoma de Baja California, Mexicali, 21000, México

**Keywords:** borderline stage, clinical obesity, hyperinsulinemia, hypertriglyceridemia, insulin resistance, low-grade inflammation, obesity

## Abstract

Obesity is a multifactorial health condition influenced by genetic predisposition and environmental factors, identified as a condition of persistent, mild systemic inflammation marked by an abnormal buildup of fat tissue, becoming clinical when accompanied by functional impairment of organs. This review explores the role of hyperinsulinemia and hypertriglyceridemia in driving the transition from a preclinical to a clinical state of obesity. Insulin resistance leads to compensatory hyperinsulinemia, impairing glucose homeostasis in skeletal muscle, liver, and adipose tissue. Concurrently, excessive dietary fat intake contributes to elevated triglyceride levels, which promote systemic inflammation and facilitate the onset of endocrine and cardio vascular disorders. Early risk factors, such as childhood obesity, as well as other contributors, including chronic psychological stress, alterations in gut microbiota, sleep disturbances, and vitamin D deficiency, are discussed in the context of their role in disease progression. Critically, the concept of a ‘borderline’ stage is introduced–a transitional phase characterized by elevated triglycerides, insulin resistance, and low-grade chronic inflammation–representing a critical point in the progression toward clinical obesity. Identifying this intermediary stage, even present in other pathologies, offers a valuable window for early interven tion, potentially preventing the establishment of chronic degenerative diseases associated with advanced obesity. Current strategies aimed at controlling hyperinsulinemia and hypertriglyceridemia, including dietary interventions, physical activity, and pharmacological approaches such as GLP-1 receptor agonists and SGLT2 inhibitors, should be considered.

## Introduction

Obesity is increasingly recognized as a stage of chronic, low-grade systemic inflammation characterized by excessive accumulation of adipose tissue. This condition arises from complex interactions among physiological, genetic, environmental, and psychosocial factors [1]. According to the World Health Organization, the global prevalence of obesity has tripled over the past five decades, and the Global Nutrition Report estimated that in 2017, approximately 41 million children under the age of five and 1.93 billion adults over 18 were overweight (BMI≥25 kg/m²), of whom 641 million met the criteria for obesity (BMI≥30 kg/m²). These data indicate that nearly 40% of the global adult population was overweight, and over 10% were obese [1]

By 2022, approximately one in eight individuals worldwide was living with obesity [2–4]. In the United States, adult obesity affects around 40% or more of the population, whereas in Mexico and several South American countries, it ranges roughly between 30% and 40% [5,6].

**Figure 1:**
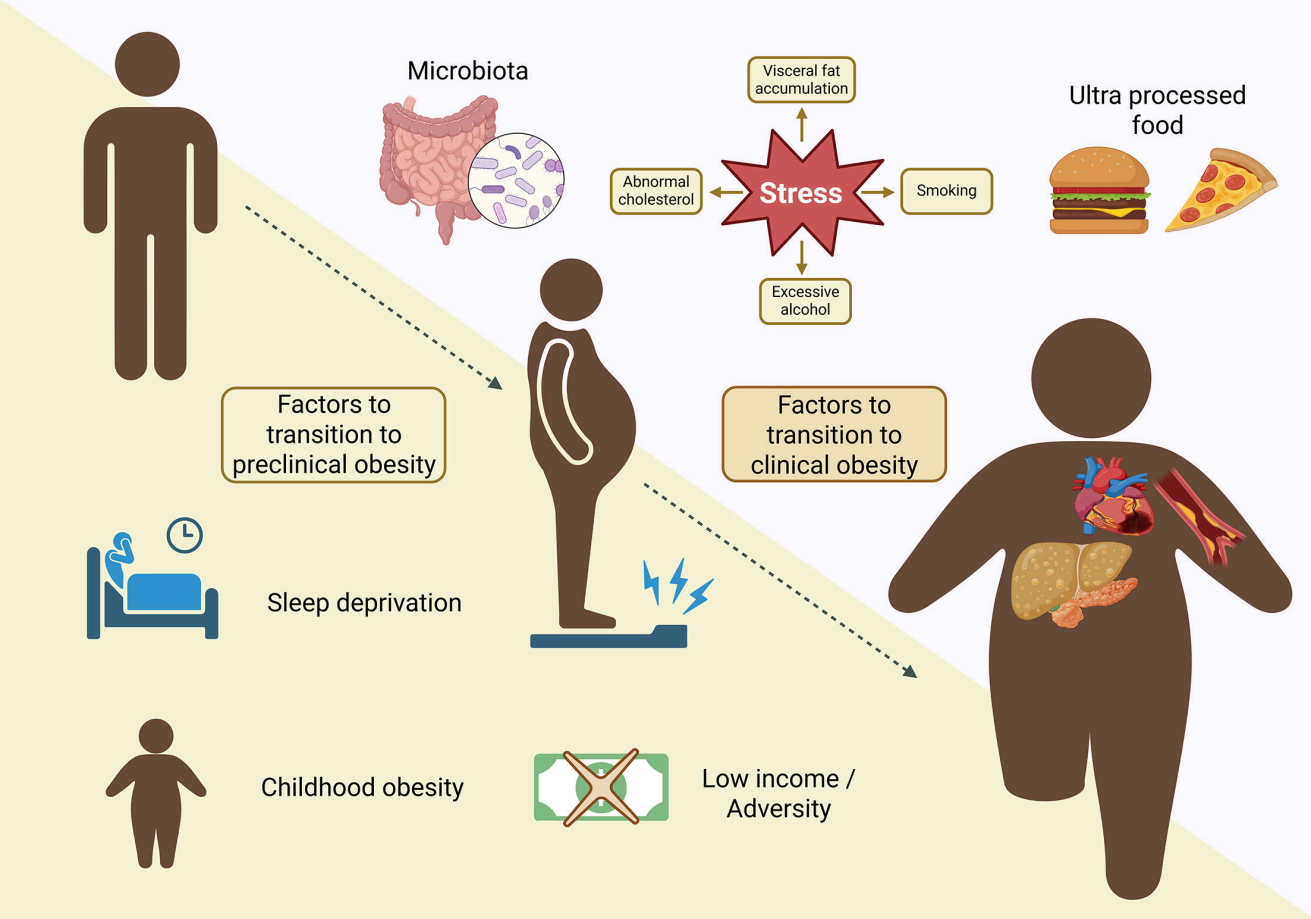
Several factors influence the development of obesity. Description of factors involved in the transition of preclinical obesity, such as sleep deprivation, childhood obesity, low income, and other factors that aggravate the state and develop the clinical state of obesity.

Recent frameworks, such as those proposed by the *Lancet Commission on Diabetes and Endocrinology*, have introduced a more nuanced classification of obesity that distinguishes between preclinical and clinical stages. This approach emphasizes the assessment of an individual’s metabolic status, highlighting factors such as hypertriglyceridemia and hyperinsulinemia as central mechanisms in the progression toward chronic degenerative diseases that ultimately affect the subject’s quality of life. These metabolic disturbances are associated with a significant decline in health-related quality of life [7]. However, the conceptual division between preclinical and clinical obesity remains subject to differing interpretations, potentially leading to the misconception that early stages maintain minimal health risks (Figure 1).

## Conditions that determine the transition to preclinical and clinical obesity

Well-established risk factors for obesity—such as physical inactivity, genetic predisposition, dyslipidemia, and elevated body mass index (BMI), particularly in individuals with excess adipose tissue, remain critical contributors to its global burden [8]. According to the Global Burden of Disease (GBD) study, in 2021, high BMI accounted for approximately 129 million disability-adjusted life years (DALYs) (95% uncertainty interval: 56.0–202 million) and 3.71 million deaths worldwide (95% UI: 1.85–5.66 million). In that year, it was ranked as the seventh-leading level 2 risk factor for DALYs [9].

### Genetics

As early as 2005, obesity had been associated with mutations in more than 600 genes or chromosomal loci [10]. From an etiological perspective, obesity can be classified as syndromic, monogenic, polygenic, or multifactorial, with heritability estimates ranging from 40 to 70% [11]. The primary genes implicated in monogenic forms of obesity include the leptin gene (LEP), the leptin receptor (LEPR), carboxypeptidase E, agouti-related peptide (AGRP), prohormone convertase 1, and pro-opiomelanocortin (POMC). Mutations in genes encoding the melanocortin receptors 3 and 4 (MC3R and MC4R) have been identified, which play a central role in regulating hunger, satiety, and energy balance. These genes also participate in adipocyte growth and differentiation, positioning them as key contributors to the early stages of obesity pathophysiology [10]. In Alström syndrome, Cohen syndrome, and Bardet-Biedl syndrome, obesity is part of the clinical phenotype associated with specific genetic mutations, characterized by early-onset obesity, typically beginning in childhood [12,13].

Beyond monogenic and syndromic forms, polygenic obesity, resulting from the cumulative effect of many small-effect variants, accounts for the vast majority of obesity cases. Twin, family, and adoption studies estimate the heritability of BMI (and obesity) to lie between 40% and 70%, illustrating a strong genetic predisposition to higher adiposity [14–17]. Among identified loci, variants in the FTO gene (Fat mass and obesity associated gene) remain the most consistently associated with higher BMI. Carriers of risk alleles (e.g., rs9939609) have a 1.6-fold increased odds of obesity and may consume ~ 125–280 kcal/day more than noncarriers [18]. FTO encodes an N6-methyladenosine (m6A) demethylase, an enzyme involved in epigenetic regulation through the removal of methyl groups from RNA. This affects mRNA splicing, stability, and translation—particularly in genes regulating energy homeostasis and adipogenesis. These molecular actions explain how FTO variants can influence appetite control, energy intake, and fat accumulation [19]. Moreover, genome-wide association studies (GWAS) have cataloged hundreds of loci associated with obesity [20]. While each contributes marginally, their collective impact aids in understanding the genetic architecture of obesity. This evidence has paved the way for polygenic risk scores (PRS)—quantitative measures aggregating multiple genetic variants to predict obesity risk. A recent PRS developed using data from over 5 million individuals explained up to 17.6% of BMI variation in populations of European ancestry [21].

However, PRS accuracy varies across ethnicities, emphasizing the urgency for ancestry-specific adjustments. For instance, in Indonesia, a PRS adjusted according to population structure improved obesity risk prediction (achieving ~ 5% higher AUC) compared with the unadjusted version [22]. 

### Obesity at early ages

Childhood obesity is a well-established predictor of adult obesity, with evidence indicating that excess weight gained during prepubertal or pubertal stages significantly increases the likelihood of persistent obesity in later life [23]. These findings suggest that early-onset obesity should not be considered a transient condition, but rather a critical determinant of long-term metabolic health.

An extensive prospective cohort study involving 62,565 Danish men demonstrated that being overweight at any point during childhood or adolescence was positively associated with an elevated risk of developing type 2 diabetes in adulthood. Underscoring the importance of early intervention to mitigate long-term metabolic consequences [24].

A systematic review and meta-analysis were conducted to evaluate the effectiveness of simple measures of childhood obesity, particularly BMI, in predicting future obesity. The review included 15 prospective cohort studies, which collectively involved 200,777 participants followed from childhood into adulthood [25]. BMI was the most commonly reported measure across all studies. The findings revealed that children and adolescents with obesity were approximately five times more likely to remain obese in adulthood compared with their normal-weight peers. Specifically, about 55% of obese children remained obese in adolescence, 80% continued to be obese in adulthood, and 70% remained so after the age of 30 [25,26].

The pathophysiological mechanisms linking childhood obesity to adult metabolic disorders involve sustained adipose tissue expansion and the early establishment of detrimental dietary and behavioral patterns. Prolonged exposure to an obesogenic environment during critical developmental windows induces metabolic adaptations, including reduced insulin sensitivity, dysregulated leptin signaling, and impaired pancreatic β-cell function [27]. Likewise, children born with macrosomia—particularly those with a birth weight exceeding 4.5 kg—have been shown to exhibit a 5.6% increased risk of developing obesity during adolescence [28].

### Association with chronic stress and influence of the social environment

Chronic stress is a well-documented factor contributing to the development and progression of obesity through several physiological and behavioral mechanisms. Stress activates the hypothalamic–pituitary–adrenal (HPA) axis, leading to increased secretion of glucocorticoids (GCs) and catecholamines, as well as stimulation of proinflammatory pathways. This neuroendocrine response promotes visceral adiposity, impairs insulin sensitivity, and alters glucose and lipid metabolism. Prolonged metabolic dysregulation under chronic stress conditions can result in β-cell dysfunction and accelerate the onset of insulin resistance and other components of metabolic syndrome [29–31].

Chronic stress influences eating behavior by increasing the consumption of hypercaloric and ultra-processed foods, often rich in fat and sugar. This phenomenon, frequently referred to as ‘emotional eating’ or ‘hedonic eating’, activates reward pathways in the brain, particularly those involving dopaminergic circuits, similar to patterns observed in substance use disorders [32].

The social environment also plays a significant role in the prevalence of obesity. Children exposed to parental obesity are at increased risk of developing obesity themselves. Factors such as parental smoking, single parenthood, low socioeconomic status, limited physical activity (particularly in boys), and excessive screen time (especially in girls) have been associated with higher obesity rates [33].

Psychosocial adversity—such as economic hardship, parental separation, chronic illness within the family, or exposure to mental health and substance use issues—has also been linked to the development of obesity and type 2 diabetes mellitus (T2DM). A longitudinal study of 4860 individuals followed from adolescence to adulthood found that those exposed to high levels of adversity had a significantly increased risk of developing clinical obesity and T2DM compared with those in low adversity settings, with both men and women showing elevated susceptibility [34]. Additional evidence supports the association between lower socioeconomic status and higher obesity prevalence across populations. In a pooled analysis of seven European birth cohorts, children of mothers with low education had a nearly 3-fold higher risk (RR = 2.99, 95 % CI 2.07–4.31) of developing obesity, and those from low-income households had RR = 2.69 (95 % CI 1.68–4.30) [35]. In U.S. adults, data from 2011 to 2014 show that obesity prevalence among women with a college education is markedly lower (27.8%) compared with those with a high school diploma or less (45.3%; *P* < 0.001) [36]. A systematic review of European studies found that lower socioeconomic status is consistently associated with less favorable dietary patterns, which are in turn linked to weight gain and obesity [37]. At the same time, lower income levels have been consistently linked to higher obesity prevalence across multiple studies and settings [38,39]. In pediatric populations, household poverty has been repeatedly associated with increased risk of overweight and obesity in industrialized countries [40].

### The role of the microbiota

The gut microbiota plays a critical role in host metabolism and immune regulation, with systemic implications that influence overall health and quality of life. Dysbiosis—an imbalance in the microbial composition, characterized by reductions in Firmicutes and Clostridia—has been linked to an obesity-related microbial profile. These alterations impair nutrient digestion and absorption, increase caloric extraction from food, promote fat accumulation, and contribute to the progression of obesity [41]. Recent studies using Mendelian randomization (MR) analyses have strengthened the evidence for a causal relationship between gut microbiota composition and metabolic disorders. Two-sample MR approaches have identified specific bacterial genera causally associated with metabolic syndrome and type 2 diabetes, suggesting that dysbiosis contributes directly to insulin resistance and glucose dysregulation [42,43].

In a cohort study of 893 individuals, significant associations were observed between gut microbiota composition and variations in BMI and lipid profiles, independent of age, sex, and genetic background [44]. Moreover, dietary composition plays a key role in shaping the microbiota. High dietary fat intake modifies gut microbial populations and promotes low-grade systemic inflammation, further contributing to the pathogenesis of obesity [45].

Several observational studies link specific microbial profiles to BMI, lipids, and glucose, but these connections may be influenced by diet and other variables. Moreover, experimental models, through gut ecosystem manipulation and microbial community transplantation between animals, have shown that transferring obesity-associated microbiota to germ-free recipients leads to increased adiposity compared with lean microbiota [46]. Clinical trials indicate that fecal microbiota transplantation (FMT) from lean donors can temporarily enhance insulin sensitivity in people with metabolic syndrome, though results vary [46–48]. This suggests that the gut microbiota not only changes with obesity but may also play a role in its progression.

A growing body of evidence supports the role of the gut microbiota in cardiometabolic diseases. A meta-analysis demonstrated that probiotic supplementation significantly reduced systolic blood pressure by 3.56 mmHg and diastolic pressure by 2.38 mmHg, underscoring the impact of microbiota composition on cardiovascular health [49]. Furthermore, the metabolism of red meat, seafood, and eggs, rich in phosphatidylcholine and L-carnitine, by gut microbes leads to the production of trimethylamine (TMA), which is subsequently converted in the liver to trimethylamine N-oxide (TMAO), a compound associated with increased atherosclerotic risk [50].

In individuals with clinical obesity, a marked reduction in Firmicutes and Clostridia, along with an increase in Betaproteobacteria, has been observed. The abundance of Betaproteobacteria positively correlates with plasma glucose levels. Higher Bacteroidetes to Firmicutes ratios and increased *Bacteroides-Prevotell*a to *C. coccoides–E. rectale* ratios have also been connected to elevated plasma glucose, suggesting a potential connection between gut microbial composition and the development of T2DM [51].

Experimental models have further supported this relationship. In obese mice, prebiotic administration resulted in a reduction of levels of plasma lipopolysaccharide (LPS), proinflammatory cytokines, and hepatic oxidative stress markers. These effects were associated with improved intestinal barrier function, reduced permeability, and enhanced tight junction integrity. Additionally, prebiotic treatment was found to stimulate endogenous glucagon-like peptide 2 (GLP-2) secretion, contributing to gut barrier maintenance and overall metabolic improvement [52].

Some interventions limiting hepatic triglyceride accumulation have shown protective effects against metabolic dysregulation. For instance, leptin has been shown to reduce hepatic lipid deposition and inflammation via activation of the JAK2-STAT3/AMPK pathway [53]. In human liver-derived organoid models, exposure to mitochondrial poisons that provoke lipid accumulation results in metabolic disturbances, illustrating how increased triglyceride burden impairs metabolic homeostasis [54]. Additionally, propionate enhances fatty acid oxidation by up-regulating genes regulated by peroxisome proliferator-activated receptor alpha (PPARα), including CPTII and TFPα [55]. Accordingly, a shift in gut microbiota composition, as commonly observed in obesity, may lead to reduced SCFA production, impaired lipid regulation, and increased susceptibility to hepatic steatosis and insulin resistance [56–58]. These findings highlight that microbial dysbiosis is not only a consequence but also a potential driver of obesity and its metabolic complications. And therefore, a condition that must be altered in the borderline stage.

### Sleep pattern and obstructive sleep apnea

Altered sleep patterns—including sleep deprivation and obstructive sleep apnea (OSA)—have been associated with the development and progression of clinical obesity, insulin resistance, and cardiovascular disease. These associations underscore the importance of maintaining adequate sleep duration and quality as essential components of metabolic health [59].

Sleep deprivation has been shown to increase appetite, reduce energy expenditure, and disrupt neuroendocrine regulation; these conditions contribute to weight gain and facilitate the transition from preclinical to clinical obesity [59].

A comprehensive meta-analysis encompassing 36 studies and a combined total of 634,511 individuals (30,002 children and 604,509 adults) reported a significant association between short sleep duration—defined as less than 5 hours per night—and an increased risk of obesity. The odds ratio (OR) for obesity was 1.89 in children and 1.55 in adults. Furthermore, adults who slept 5 h or fewer per night had a markedly elevated risk of obesity. In contrast, each additional hour of sleep was associated with a 0.35 kg/m² reduction in body mass index (BMI) [60].

Supporting this evidence, a prospective meta-analysis involving over 480,000 participants, with follow-up periods ranging from 2.5 to 16 years, identified 8443 new cases of type 2 diabetes. The lowest risk was observed in individuals who slept between seven and eight hours per night. Compared with those who slept seven hours, each hour of reduced sleep increased the risk of diabetes by 9%, whereas each additional hour beyond seven was associated with a 14% increase in risk [61].

Additionally, a meta-analysis of six prospective cohort studies, including 5953 participants with follow-ups ranging from 2.7 to 16 years, identified 332 new cases of T2DM. The findings indicated that individuals with moderate-to-severe OSA had a 65% higher risk of developing T2DM compared with those without OSA [62]. This elevated risk is attributed to intermittent hypoxia, increased oxidative stress, and fragmented sleep. These alterations promote sympathetic nervous system activation, systemic inflammation, and impaired glucose metabolism, ultimately contributing to the development of insulin resistance and the onset of T2DM [63].

### The impact of Vitamin D

Vitamin D deficiency has been linked to an increased risk of obesity, likely due to its role in energy metabolism, adipose tissue function, and inflammation. Experimental studies have shown that vitamin D inhibits adipogenesis by down-regulating key transcription factors, including C/EBPα, PPARγ, C/EBPβ, and adipocyte protein 2 (AP2). During the final stages of adipocyte differentiation, C/EBPβ and C/EBPδ induce the expression of C/EBPα and PPARγ, promoting the maturation of preadipocytes into adipocytes. AP2, also known as fatty acid-binding protein 4, facilitates the intracellular transport of fatty acids toward the PPARγ receptor in the nucleus, enhancing its transcriptional activity. Inhibition of these pathways suggests that vitamin D may interfere with the terminal differentiation of 3T3-L1 adipocytes [64].

In clinical settings, evidence regarding vitamin D supplementation remains controversial. A meta-analysis of five trials involving 1080 participants reported that 21.2% of individuals receiving vitamin D achieved reversion from prediabetes to normoglycemia, compared with 14.1% in the control group, with some subgroups reaching rates as high as 48% [65]. However, a separate meta-analysis of ten randomized controlled trials found no significant improvements in insulin resistance or 2 h plasma glucose levels during oral glucose tolerance testing, with mean differences of −0.06 and −0.23 mmol/l, respectively [66].

Beyond its metabolic effects, vitamin D is extensively stored in adipose tissue, primarily in its native forms (D₂ and D₃) and its circulating metabolite 25-hydroxyvitamin D (25(OH)D), with approximately 65% of the vitamin D retained unmodified. In individuals with obesity, the relationship between adipose tissue stores and circulating 25(OH)D levels is complex and multifaceted. One study found that, following Roux-en-Y gastric bypass surgery, vitamin D concentrations were ~20% higher per gram in visceral tissue compared with subcutaneous adipose tissue [67]. Although serum vitamin D levels are commonly used as a surrogate for total body stores, recent evidence suggests that distribution and bioavailability vary across fat depots—particularly between the subcutaneous and visceral compartments—which may limit the accuracy of serum levels in reflecting systemic vitamin D status in obese individuals [68].

Several studies across age groups and populations have consistently identified an inverse association between serum vitamin D levels and obesity. In children and adolescents, higher vitamin D concentrations are associated with a lower prevalence of abdominal obesity, indicating a dose-response relationship. In older adults, serum 25(OH)D levels correlate negatively with BMI, waist circumference, and total body fat mass [69,70].

Findings from animal models further support these observations. In rodents with diet-induced obesity, high-fat diets disrupt vitamin D homeostasis, potentially through alterations in the expression of metabolic enzymes in the liver and kidneys. Vitamin D deficiency in these models is associated with worsened insulin resistance, hepatic steatosis, and systemic inflammation [71].

Proposed mechanisms underlying vitamin D deficiency in obesity include volumetric dilution, sequestration within adipose tissue, and reduced cutaneous synthesis due to limited sun exposure. Moreover, vitamin D appears to influence adipose tissue biology by modulating adipocyte differentiation, apoptosis, and energy metabolism, thereby reinforcing its potential role in regulating adiposity [72].

## Tissue-specific mechanisms of insulin resistance in obesity

The insulin signaling cascade begins with insulin binding to its receptor, leading to receptor dimerization and the activation of insulin receptor substrates (IRS-1 and IRS-2) via tyrosine phosphorylation [73]. This enables the recruitment of phosphoinositide 3-kinase (PI3K), which catalyzes the conversion of phosphatidylinositol-4,5-bisphosphate (PIP2) into phosphatidylinositol-3,4,5-trisphosphate (PIP3) [74], thereby facilitating Akt activation by PDK1 and mTORC2 [75,76] This pathway constitutes the central axis of insulin signaling; thus, any alteration that reduces Akt activation compromises insulin action, such as the presence of obesity. The following sections will detail the specific mechanisms contributing to insulin resistance in different tissues.

Obesity and insulin resistance are closely related through several pathophysiological mechanisms. Excess adipose tissue not only serves as an energy reservoir but also exerts endocrine functions that affect metabolic homeostasis. The increased secretion of adipokines and proinflammatory cytokines, such as tumor necrosis factor-alpha (TNF-α) and interleukin-6 (IL-6), disrupts normal insulin signaling by impairing the phosphorylation of insulin receptor substrate (IRS) [77,78]. This disruption compromises the activation of the phosphatidylinositol 3-kinase (PI3K) and Akt pathways, which are essential for glucose uptake in peripheral tissues, such as skeletal muscle [79].

In addition, endoplasmic reticulum (ER) stress and ectopic lipid accumulation in nonadipose organs activate inflammatory and cellular stress pathways, further exacerbating metabolic dysfunction. Obesity also contributes to β-cell failure through multiple interrelated mechanisms. Chronic nutrient overload and elevated circulating free fatty acids—particularly saturated fatty acids—induce lipotoxicity and oxidative stress in pancreatic islets. These insults impair mitochondrial function and ATP production, while also triggering ER stress, ultimately leading to defective insulin synthesis and secretion [80].

Furthermore, obesity-associated systemic inflammation increases local levels of cytokines such as IL-1β and TNF-α, which activate intracellular stress pathways, including the NLRP3 inflammasome and lipid-induced amyloidogenic processes, thereby exacerbating β-cell apoptosis and impairing their identity and regenerative capacity [81,82]. The combination of peripheral insulin resistance and progressive β-cell dysfunction creates a metabolic environment that, if not adequately compensated, culminates in the development of T2DM [83].

### Skeletal muscle

Skeletal muscle is characterized by glucose uptake mediated by the GLUT4 transporter, whose translocation to the plasma membrane is strictly insulin-dependent. Insulin stimulates glycogen synthesis through the activation of glycogen synthase, both of which are essential processes for maintaining glycemic homeostasis [84,85]. In this context, the pioneering studies by Randle et al. proposed that increased fatty acid oxidation in skeletal muscle leads to elevated citrate levels. This metabolite inhibits phosphofructokinase-1, thereby reducing glycolysis and glucose uptake by muscle cells [86].

More recent studies have identified the accumulation of lipid metabolites, such as diacylglycerols (DAG) and ceramides, as a key factor in skeletal muscle insulin resistance and increased obesity. These metabolites activate protein kinase C theta (PKCθ), which promotes the phosphorylation of IRS1 on serine residues instead of tyrosine, thereby interfering with its activation and impairing insulin signaling efficiency [87,88]. Similarly, the PKCε isoform is elevated in the skeletal muscle of insulin-resistant patients; however, its specific role in IRS1 modulation in this tissue remains unclear, unlike its well-established mechanism in hepatocytes [89].

**Figure 2:**
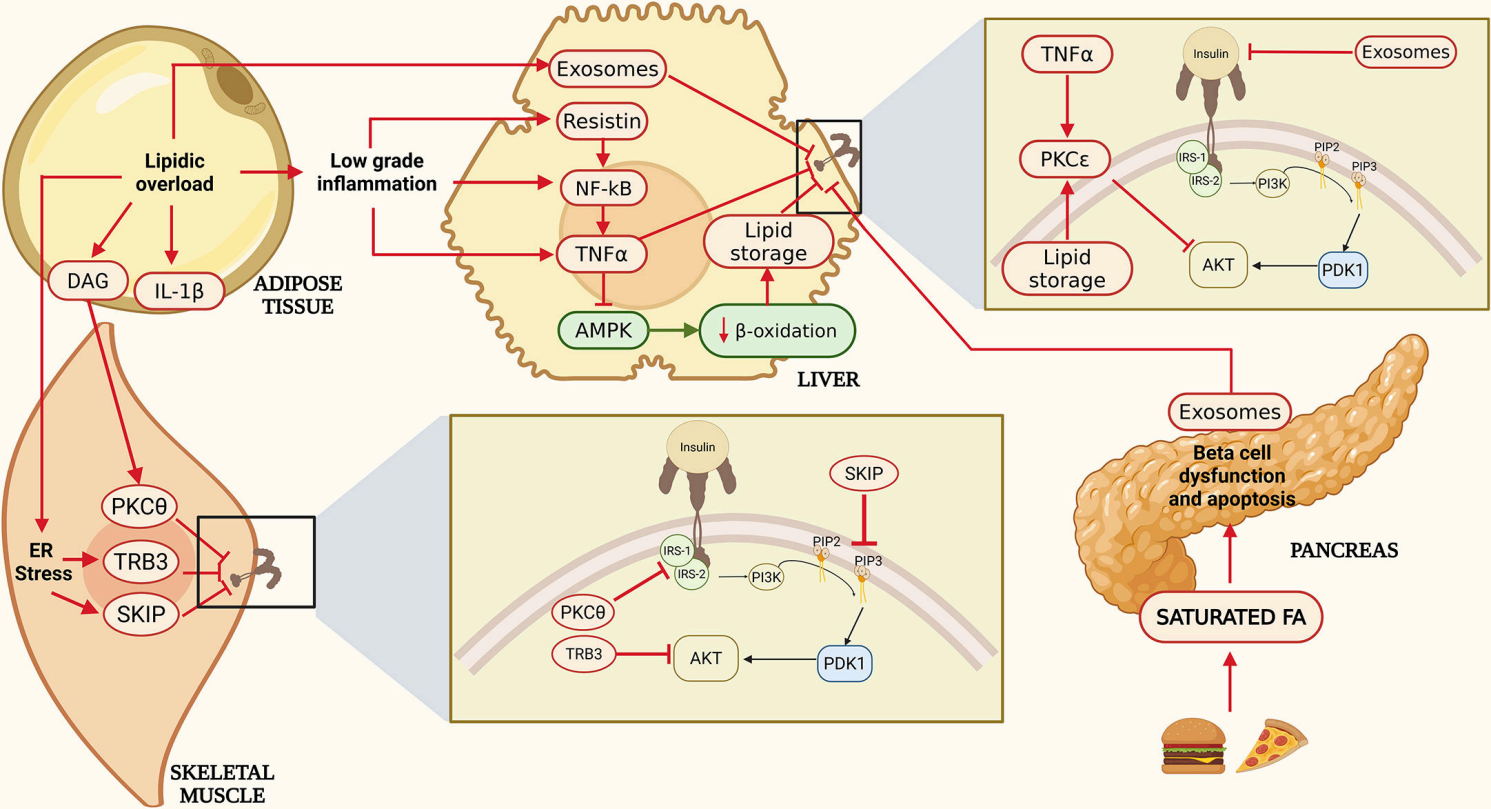
Mechanisms of insulin resistance across metabolic tissues. Lipid overload and chronic overnutrition lead to the accumulation of diacylglycerols (DAG), proinflammatory cytokines (e.g., IL-1β, TNF-α), and endoplasmic reticulum (ER) stress, which activate PKC isoforms (θ and ε), TRB3, and SKIP in skeletal muscle and liver. These mediators inhibit IRS-1/2 phosphorylation and reduce PI3K–Akt signaling, impairing GLUT4 translocation and glucose uptake. In hepatocytes, resistin and TNF-α impair insulin sensitivity via NF-κB activation and suppression of AMPK activity, decreasing β-oxidation and promoting lipid storage. Inflammatory exosomes and saturated fatty acids also reach pancreatic β-cells, contributing to dysfunction and apoptosis. Together, these inter-organ mechanisms drive systemic insulin resistance and metabolic dysregulation. Adapted from [73–102]

In addition, expression of Tribbles 3 (TRB3), a pseudokinase that inhibits insulin signaling by interacting with Akt and preventing its activation, contributes to insulin resistance not only in liver but also in skeletal muscle. TRB3 mediates endoplasmic reticulum stress–induced insulin resistance in myocytes by blocking Akt phosphorylation and glucose uptake, promoting lipid accumulation and mitochondrial dysfunction. Conversely, inhibition of TRB3 restores insulin sensitivity and protects against high-fat diet–induced metabolic alterations [90,91]. ER stress activates the ATF6 and XBP1 pathways, promoting the expression of Skeletal muscle and kidney-enriched inositol polyphosphate 5-phosphatase (SKIP), a phosphatase that reduces PIP3 activity, thereby indirectly inhibiting Akt activation and impairing glucose uptake in skeletal muscle [92] (Figure 2).

#### Liver

Lipotoxicity associated with obesity is a critical factor in the development of insulin resistance, as it is linked to the ectopic accumulation of fatty acids in hepatocytes. This phenomenon may result from increased lipid intake, enhanced lipogenesis, or impaired fatty acid oxidation. Consequently, lipid metabolism intermediates, such as diacylglycerol (DAG), accumulate and disrupt insulin signaling through activation of PKCε, contributing to hepatic insulin resistance observed in nonalcoholic fatty liver disease (NAFLD) [93,94].

Unlike skeletal muscle, glucose uptake in hepatocytes is not dependent on insulin. This characteristic is attributed to the constitutive expression of the GLUT2 transporter, which facilitates the bidirectional exchange of glucose without requiring insulin-induced translocation, in contrast to the GLUT4 transporter in skeletal muscle [95]. Nevertheless, insulin plays a crucial role in regulating hepatocyte macronutrient metabolism [96], making insulin resistance a key pathophysiological factor in hepatic function.

Chronic low-grade inflammation in the liver plays a pivotal role in insulin resistance. In this context, activation of the NF-κB pathway in hepatocytes promotes the synthesis of proinflammatory cytokines, such as tumor necrosis factor-alpha (TNF-α) and interleukin-6 (IL-6), by Kupffer cells [97]. These cytokines stimulate lipolysis in adipocytes, increasing the release of free fatty acids into the liver. Notably, adipocytes can also produce these cytokines in the setting of chronic metabolic inflammation associated with obesity, where they are classified as adipokines. Among them, resistin has been specifically implicated in modulating hepatic insulin resistance [98] (Figure 2).

TNF-α has been implicated in the inhibition of AMP-activated protein kinase (AMPK) activity, leading to reduced fatty acid catabolism and, consequently, increased lipid storage in hepatocytes [99]. More recently, diet-induced obesity has been shown to enhance the secretion of adipose tissue-derived exosomes, which can induce insulin resistance through inflammatory mechanisms [100–102].

### Adipose tissue

Insulin-dependent GLUT4 mediates glucose uptake in adipose tissue similarly to skeletal muscle; however, adipose tissue contributes to less than 5% of total glucose disposal following oral intake, with skeletal muscle accounting for the majority of insulin-stimulated glucose uptake [103]. During this process, glycerol-3-phosphate is produced and utilized for triglyceride synthesis, thereby reducing lipid transport to muscle and liver. Consequently, a crucial function of insulin in adipose tissue is the suppression of lipolysis. However, the molecular mechanisms underlying adipose tissue insulin resistance are not completely elucidated [104] (Figure 2).

The molecular alterations described above illustrate how obesity induces insulin resistance through tissue-specific mechanisms affecting skeletal muscle, liver, and adipose tissue. These pathophysiological changes contribute to a progressive metabolic deterioration that, if left unaddressed, culminates in overt clinical manifestations such as T2DM and cardiovascular disease. In this context, differentiating between preclinical and clinical obesity becomes essential to identify early metabolic disturbances and implement timely interventions to prevent irreversible systemic damage.

## Preclinical obesity and clinical obesity, differences

The Lancet Commission on Diabetes and Endocrinology defines preclinical obesity as a condition characterized by excess adiposity in the absence of overt dysfunction in tissue or organ systems [7]. Despite the lack of clinical manifestations, individuals in this stage present a significantly elevated risk of developing noncommunicable diseases (NCDs), including T2DM, cardiovascular disease, and certain cancers. The diagnosis of preclinical obesity is based on body fat quantification and anthropometric measurements adjusted for sex and other individual characteristics (Table 1) [7].

**Table 1:** Main features that define preclinical obesity, borderline stage, and clinical obesity

Characteristic	Preclinical obesity	Borderline stage	Clinical obesity
BMI	25–27.9 kg/m^2^	28–31.9 kg/m^2^	> 32 kg/m^2^
Chronic inflammation	Absent or mild	Moderately elevated C-reactive protein, IL-6, and TNF	Established systemic low-grade inflammation
Dysfunction of adipose tissue	No evidence	Expansion of adipose tissue with local hypoxia	Severe adipose dysfunction
Insulin resistance	Normal	HOMA-IR moderately elevated	High HOMA-IR and hyperglycemia
Lipid profile	Normal or mild	High triglycerides, low HDL	Established dyslipidemia
Significant limitations in daily activities	Absent	Moderate functional limitation	Marked functional limitation
Intervention approach	Preventive and changes in lifestyle	Drug treatment and lifestyle changes	Therapeutic
Presence of comorbidities	Absent	Present, mainly insulin resistance	Present, type 2 diabetes mellitus, hypertension, and dyslipidemia

Modified from Figure 3 of [7].

**Figure 3:**
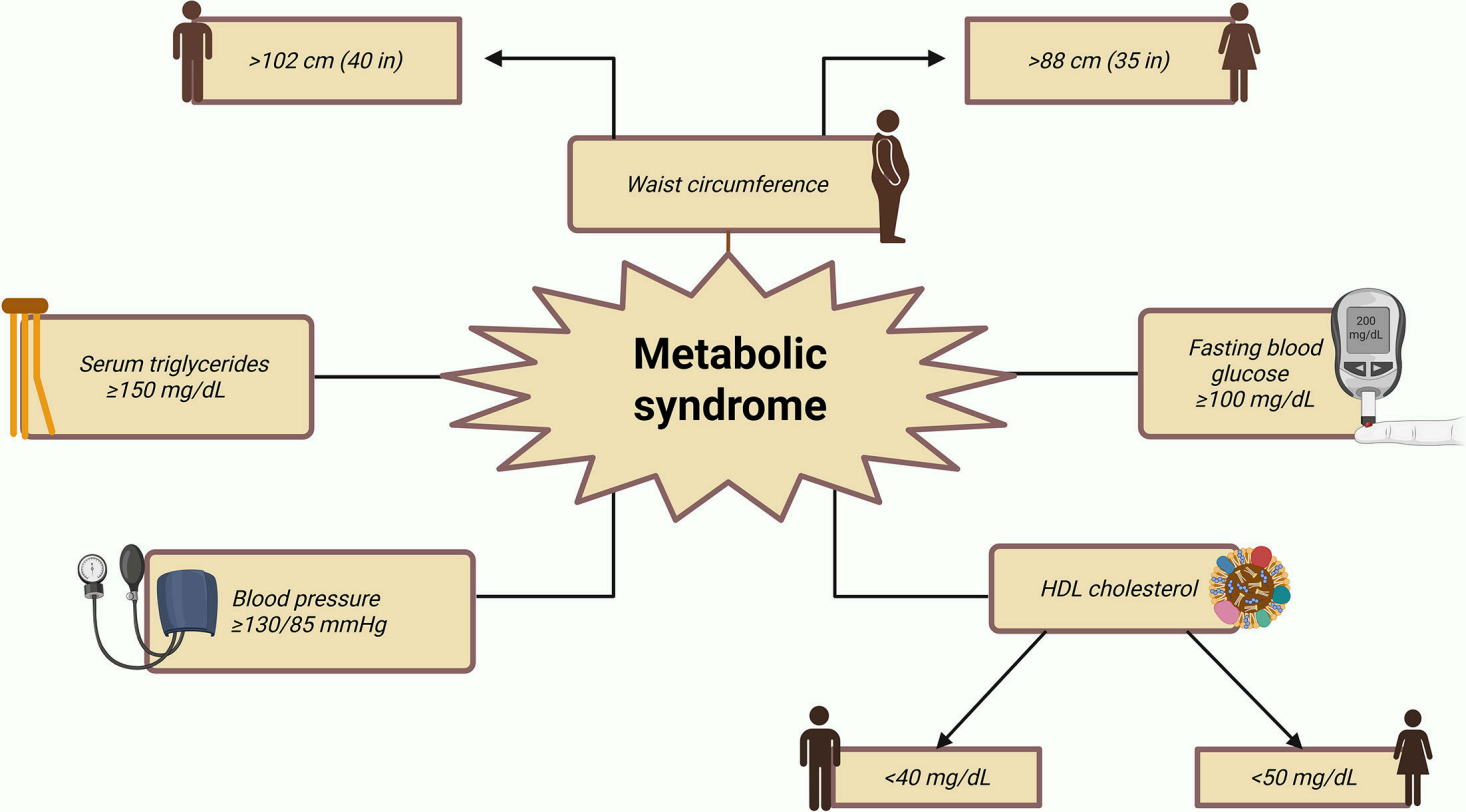
Diagram representing the criteria that comprise metabolic syndrome.

In contrast, clinical obesity is defined by the presence of excessive adiposity accompanied by functional impairment of tissues and organs, as well as limitations in performing activities of daily living (Table 1). Given the potential for disease progression and complications, all individuals with obesity—regardless of clinical stage—should receive personalized counseling, ongoing health monitoring, and targeted therapeutic interventions aimed at achieving improvement or, when feasible, remission of the disease [7]. However, based on the evidence, there is an intermediate range that is not considered, but which may be fundamental: the borderline stage.

As cited above, Table 1 illustrates the progressive transition from preclinical obesity to clinical obesity, introducing the borderline stage as an intermediate phenotype. In preclinical obesity, metabolic parameters are mostly normal, with only mild or no functional limitations, and interventions are preventive. In contrast, clinical obesity is defined by overt metabolic dysfunction, systemic inflammation, established comorbidities, and the need for therapeutic management. The borderline stage represents a critical window wherein early dysfunction begins to emerge—such as elevated HOMA-IR, mild dyslipidemia, and low-grade inflammation—without meeting full diagnostic criteria. Recognizing this stage may allow for timely interventions to prevent irreversible metabolic damage. These distinctions, outlined in Table 1, are further explored in the following sections.

Moreover, analogous to obesity, several other medical conditions are characterized by borderline or preclinical stages that precede the overt manifestation of disease. For instance, elevated blood pressure, formerly termed prehypertension, is defined as a systolic pressure of 120–129 mmHg with diastolic pressure < 80 mmHg and identifies individuals at increased risk of progressing to established hypertension [105]. In bone health, osteopenia represents a reduction in bone mineral density that is less severe than osteoporosis, yet it confers a significantly elevated risk of developing the latter condition [106]. Similarly, subclinical thyroid disorders, including both subclinical hypothyroidism and hyperthyroidism, are defined by abnormal thyroid-stimulating hormone (TSH) levels with normal circulating thyroid hormone concentrations, in the absence of overt clinical symptoms [107]. These stages are considered early indicators of thyroid dysfunction. In the gynecological domain, perimenopause (or premenopause) refers to the transition period preceding menopause, marked by endocrine alterations and the onset of early symptoms prior to the permanent cessation of menstruation [108] Moreover, in type 2 diabetes mellitus, a well-recognized precursor state, prediabetes, is characterized by blood glucose levels elevated above normal but not meeting diagnostic criteria for diabetes, representing an intermediate metabolic stage with high risk of progression [109]. These examples support the clinical relevance of identifying transitional stages across diseases, providing a conceptual framework for recognizing and managing borderline obesity.

### Borderline stage and biochemical parameters

It is essential to note that obesity, in the absence of behaviors that promote metabolic homeostasis, can serve as a precursor to its clinical manifestation. The term ‘borderline’ is a proposal employed to describe this transitional phase, characterized by the initial onset of multisystem dysfunction—metabolic, cardiovascular, endocrine, respiratory, among others—without meeting the full criteria for a chronic disease. At this stage, specific biochemical parameters may be elevated, yet remain below established diagnostic thresholds. Focusing on this subclinical state could represent a critical window for early detection and intervention.

#### Definition of the borderline stage

**Figure 4:**
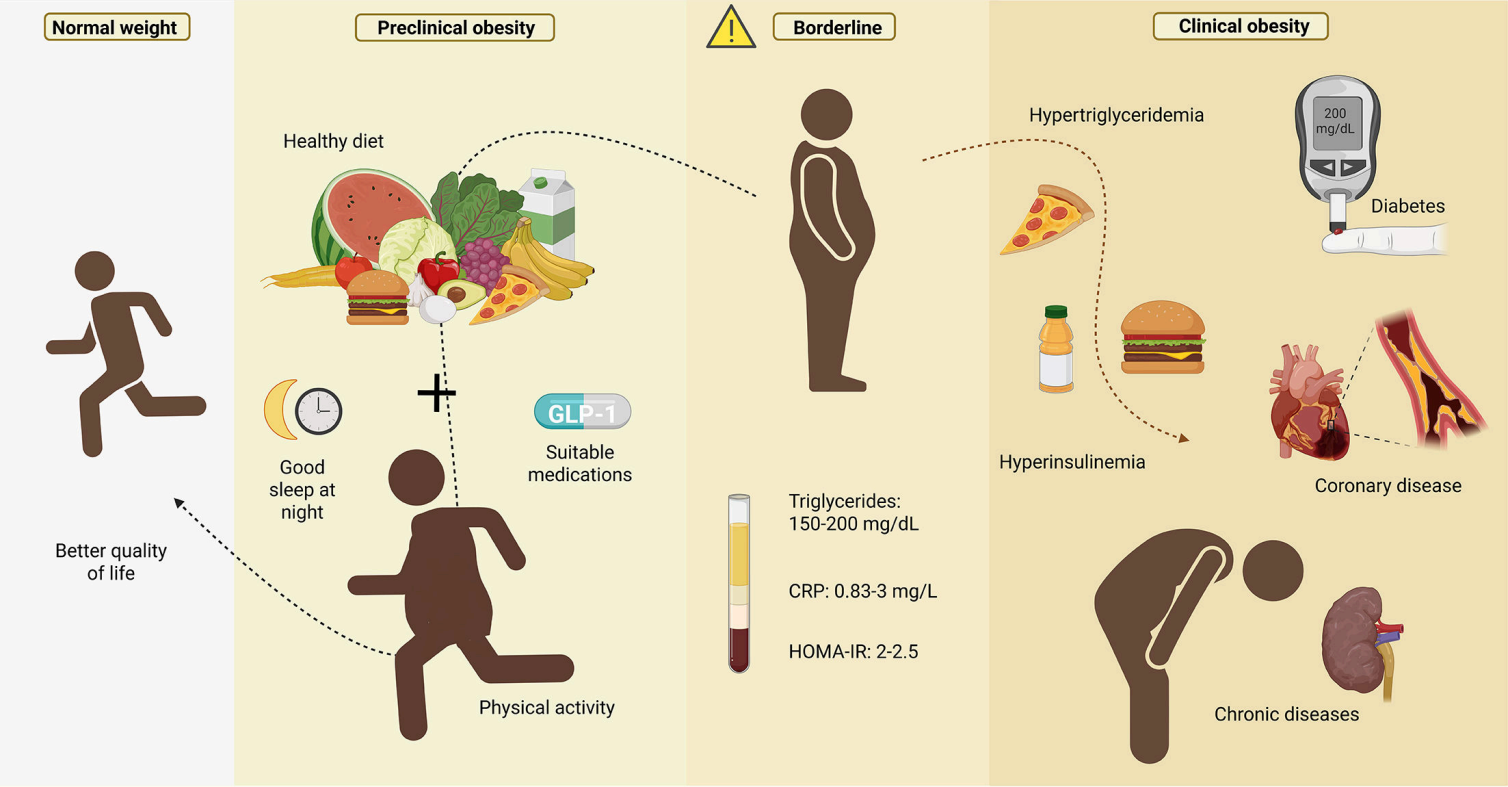
The progression from normal weight to clinical obesity is characterized by distinct stages, highlighting preventive strategies such as a healthy diet, regular physical activity, adequate sleep, and medications like GLP-1 agonists. Without intervention, individuals may reach borderline and clinical obesity, marked by hypertriglyceridemia, hyperinsulinemia, and increased risk of type 2 diabetes, coronary artery disease, and chronic illnesses. Key biomarkers in the borderline stage include triglycerides (150–200 mg/dl), C-reactive protein (0.83–3 mg/l), and HOMA-IR (2–2.5), useful for early detection and prevention.

The borderline stage refers to a transitional metabolic condition in which homeostasis is progressively disrupted due to excessive accumulation of adipose tissue. Although the degree of adiposity may not yet meet the criteria for severe obesity, early functional alterations in various systems can be observed. These include insulin resistance, endothelial dysfunction, elevated blood pressure, dyslipidemia, and low-grade systemic inflammation. Despite the presence of these early abnormalities, overt comorbidities such as T2DM or cardiovascular disease have not yet been diagnosed. Nevertheless, the risk of progression to clinical obesity is significantly increased, highlighting the importance of timely therapeutic intervention (Figure 4). In light of the progressive nature of adiposity-related dysfunction, it is reasonable to define the ‘borderline’ stage of obesity using a BMI range of 28–31.9  kg/m² (Table 1), bridging the gap between overweight and clinically defined obesity. This intermediate stage might correspond to a BMI range somewhat below the classical obesity cutoff, as metabolic dysfunctions often emerge before reaching BMI ≥ 30. Some meta-analyses suggest that individuals in the lower ranges of obesity grade 1 do not always exhibit increased mortality risk [110], supporting the notion of a ‘gray zone’ where early interventions may be particularly impactful.

### Biochemical parameters and risk classification indexes

 To expand on the concept of borderline stage, a review of the literature was conducted to identify biochemical markers that may indicate imminent progression to clinical obesity. Specific attention was given to hypertriglyceridemia, hyperinsulinemia, and low-grade inflammation as potential indicators for early identification and timely intervention (Table 1).

#### Hypertriglyceridemia

A cohort study with over 13,000 participants reported that individuals developing hypertriglyceridemia (serum triglycerides ≥ 2.3 mmol/l or ≈200 mg/dl) had significantly higher cardiovascular disease (CVD) risks: relative risk (RR) of 2.61 in those under 45 years, 1.41 in ages 45–54, and 1.30 in ages 55–64, with no significant risk increase in individuals aged 65 and older [111]. Early-onset cases were associated with unhealthy behaviors and obesity, as well as lower treatment adherence [111]. Importantly, emerging evidence indicates that CVD risk may begin at triglyceride levels below traditional thresholds. Meta-analyses suggest that cardiovascular risk elevations occur even at levels lower than 150  mg/dl[112], and that both fasting and postprandial hypertriglyceridemia independently predict atherosclerotic disease [113,114].

Moreover, a longitudinal Chinese study of young adults (mean age ~30 years) found that individuals in the highest quartile of fasting triglycerides had a 2.26-fold increased risk of CVD and a 1.61-fold higher risk of all-cause mortality over 11 years, compared with those in the lowest quartile [115,116]. Additionally, elevated triglyceride levels pose a particularly high risk in women and adults under 50 years, even in the absence of other traditional risk factors [117].

Based on the cumulative evidence, it is prudent to consider triglyceride levels ≥ 200  mg/dl as indicative of imminent vascular risk, while levels in the range of 140–199  mg/dl may constitute a ‘borderline’ risk zone, demanding closer monitoring and early interventions.

#### Hyperinsulinemia

Hyperinsulinemia is closely associated with the degree of insulin resistance (IR), and its assessment—alone or in conjunction with glucose levels—is commonly used to estimate metabolic dysfunction, based on the mechanisms described in section 3. Accurate quantification of IR is essential for diagnosis, treatment monitoring, prognosis, and evaluating pharmacological interventions [118].

Several longitudinal studies have demonstrated its predictive value for future metabolic alterations. The IDEFICS study (*Identification and prevention of dietary- and lifestyle-induced health effects in children and infants*), conducted in a European pediatric cohort (*n* = 3,348), reported that increases in adiposity and waist circumference were prospectively associated with higher HOMA-IR values. In contrast, reductions in HOMA-IR over time corresponded with improvements in body composition [119]. In adults, the Jackson Heart Study showed that participants with both obesity and insulin resistance (HOMA-IR ≥ 2) had a significantly higher risk of developing type 2 diabetes (IRR = 2.35) compared with those without these conditions, underscoring the utility of HOMA-IR for risk stratification beyond BMI [120–122]. More recently, the Qatar Biobank established population- and sex-specific cut-off values for HOMA-IR, highlighting the importance of contextualizing diagnostic thresholds in relation to demographic characteristics [121].

The most widely used method to estimate IR is the Homeostasis Model Assessment of Insulin Resistance (HOMA-IR), calculated using the following formula, *HOMA-IR = (Fasting insulin (μU/ml) x Fasting glucose (mmol/l))/22.5*. Alternative indexes such as the Quantitative Insulin Sensitivity Check Index (QUICKI) have also been proposed. In a study by [122], the HOMA-IR index was evaluated using a single determination of fasting insulin, demonstrating a sensitivity of 83–100% and a predictive value of greater than 89%. However, a threshold of ≥ 3.2 was suggested to risk false negatives in individuals with borderline insulin levels [122]. To date, no universal cut-off value has been established due to population variability (age, sex, ethnicity, and family history). Nonetheless, several studies have proposed that a HOMA-IR value greater than 2.6 is indicative of insulin resistance [123,124].

Insulin resistance is a hallmark of metabolic syndrome, a chronic and progressive inflammatory condition characterized by the coexistence of at least three of the following criteria [125] (Figure 3).

The central feature of clinical obesity is the excessive accumulation of adipose tissue, which promotes the secretion of proinflammatory cytokines and adipokines that interfere with insulin signaling. Tumor necrosis factor-alpha (TNF-α) impairs insulin action through serine phosphorylation of the insulin receptor substrate-1 (IRS-1), thereby attenuating downstream signaling. Furthermore, adipocyte hypertrophy induces local hypoxia, oxidative stress, and endoplasmic reticulum (ER) stress, all of which activate the c-Jun N-terminal kinase (JNK) and IKKβ/NF-κB pathways, amplifying inflammation and disrupting insulin sensitivity. These mechanisms establish a clear pathophysiological link between adiposity and insulin resistance, reinforcing the need for accessible, cost-effective tools for early identification of individuals in the borderline metabolic stage [126–129].

Likewise, a longitudinal study involving 4954 nondiabetic adults evaluated the association between changes in fasting insulin and the future development of non-alcoholic fatty liver disease (NAFLD) over five years. The results showed that individuals with elevated insulin at follow-up had a significantly higher risk of developing NAFLD, regardless of their baseline insulin levels [130]. These findings support considering a HOMA-IR range of 2.0–2.5 as a potential borderline zone for chronic disease risk.

### Low-grade inflammation markers

A meta-analysis examined differences in inflammatory markers such as C-reactive protein (CRP), IL-6, and tumor necrosis factor-alpha (TNF-α) across various metabolic obesity phenotypes. It included 91 studies reporting data on 435,007 individuals. These included metabolically healthy non-obese (MHNO), metabolically healthy obese (MHO), metabolically unhealthy non-obese (MUNO), and metabolically unhealthy obese (MUO) individuals [131]. CRP levels in the MHO group were significantly higher than those observed in MHNO individuals. The standardized mean difference (SMD) was 0.63 (95% confidence interval (CI): 0.49–0.76), while the absolute mean difference (MD) was 0.83 mg/l (95% CI: 0.56–1.11). CRP levels in MHO were also higher than those in MUNO individuals, but lower than in MUO participants. Based on these findings, a CRP level around 0.83 mg/l could be considered a potential threshold between a metabolically healthy and an at-risk inflammatory state.

Similarly, IL-6 levels in MHO individuals were higher than those in MHNO but lower than those observed in MUO subjects. TNF-α concentrations also followed this pattern, being elevated in MHO compared with MHNO, further indicating the presence of low-grade inflammation even in metabolically healthy obese individuals [131].

Furthermore, recent work indicates that CRP concentrations are elevated in metabolically unhealthy individuals even in the absence of clinical obesity, underscoring its utility as a marker of subclinical metabolic dysfunction. In a population-based analysis [132], examined low-grade systemic inflammation—quantified by hs-CRP—in 21,112 Korean adults from KNHANES (2015–2018). Median hs-CRP was 0.54 mg/l overall and varied by metabolic–obesity phenotype: 0.38 (MHNO), 0.51 (MUNO), 0.61 (MHO), and 0.80 mg/l (MUO), displaying a stepwise pattern with worsening metabolic health and adiposity. Among men, medians were 0.40 (MHNO), 0.60 (MUNO), 0.60 (MHO), and 0.80 mg/l (MUO); among women, 0.35 (MHNO), 0.50 (MUNO), 0.63 (MHO), and 0.85 mg/l (MUO). The correlation between adiposity and inflammation was stronger in women (*r* = 0.27) than in men (*r* = 0.15). After multivariable adjustment, hs-CRP was higher relative to MHNO by 29.3% (MUNO), 15.4% (MHO), and 23.3% (MUO); within obesity, MUO showed an additional 14.1% increase versus MHO. Sex-stratified models revealed larger effects in women (MUNO + 30.2%, MHO + 16.0%, MUO + 22.8%) than in men (MUNO + 22.3%, MHO + 15.8%, MUO + 12.5%); the MUO–MHO contrast was significant in women (+38.7%) but not in men. Collectively, these data argue that ‘metabolically healthy obesity’ is not inflammation-free and that hs-CRP captures subclinical risk even among normal-weight individuals with metabolic dysfunction [132].

Therefore, borderline-range levels would fall within 0.83 mg/l to 3 mg/l for CRP and between 1 and 2.5 pg/ml for IL-6. Further studies are needed to establish a proposed threshold for TNF-α; nonetheless, a range of 2.0–4.0 pg/ml could be considered a moderate risk. Establishing that ‘healthy obese’ individuals are about to progress to clinical obesity.

### The prediabetes term as a comparative model

The term prediabetes is widely used yet remains imprecise and somewhat arbitrary. Since its introduction, the validity of this approach has been debated due to diagnostic criteria that do not consistently predict progression to T2DM. Prediabetes is typically defined by impaired fasting glucose (IFG; 100–125 mg/dl), impaired glucose tolerance (IGT; 140–199 mg/dl at two hours), and/or elevated glycated hemoglobin (HbA1c; 5.7–6.4%) [133]. Rather than representing a distinct clinical entity, prediabetes should be understood as part of a continuum of dysregulated glucose metabolism; by itself, it does not encompass the altered metabolic processes in a patient with abnormal lab results and their associated morbidity risk.

More than a mere transitional phase, prediabetes reflects an early manifestation of metabolic dysfunction and serves as a significant predictor of future cardiometabolic disease [134]. In a similar vein, the concept of borderline stage seeks to capture an early and subclinical stage of pathophysiological change. However, unlike prediabetes, which focuses on glucose metabolism, the borderline framework encompasses a broader range of metabolic alterations—including lipid dysregulation, systemic inflammation, and mitochondrial impairment. Recognizing these early changes provides an opportunity for timely intervention before irreversible clinical disease develops.

### Pathophysiological processes prior to glucose alterations

A glucose-centered diagnostic approach may overlook critical metabolic alterations that precede detectable changes in blood glucose levels. Insulin resistance, endothelial dysfunction, and chronic low-grade inflammation often develop in the early stages of metabolic dysregulation—well before hyperglycemia or elevated HbA1c levels are apparent.

In obesity, multiple insulin-sensitive organs, including adipose tissue, liver, skeletal muscle, and pancreas, undergo inflammatory changes. These tissues exhibit macrophage infiltration and a shift toward pro-inflammatory immune cell phenotypes, leading to an increased production of cytokines that interfere with insulin signaling pathways. This inflammatory milieu not only exacerbates peripheral insulin resistance but also promotes β-cell dysfunction [135].

Experimental studies have demonstrated that insulin receptor (IR) expression is significantly reduced in the white adipose tissue (WAT) of db/db mice and animals fed a high-fat diet (HFD). Similar reductions in IR expression have been observed in visceral adipose tissue of obese humans, as well as in the liver and skeletal muscle of db/db and HFD-fed mice. Given that decreased IR expression impairs insulin signaling and metabolic function, these findings suggest a key mechanism by which obesity contributes to systemic insulin resistance [136]. These processes are strongly associated with visceral adiposity, which plays a central role in the development of metabolic syndrome and insulin resistance, even in the absence of overt glucose abnormalities.

### Mitochondrial dysfunction in the borderline stage

**Figure 5:**
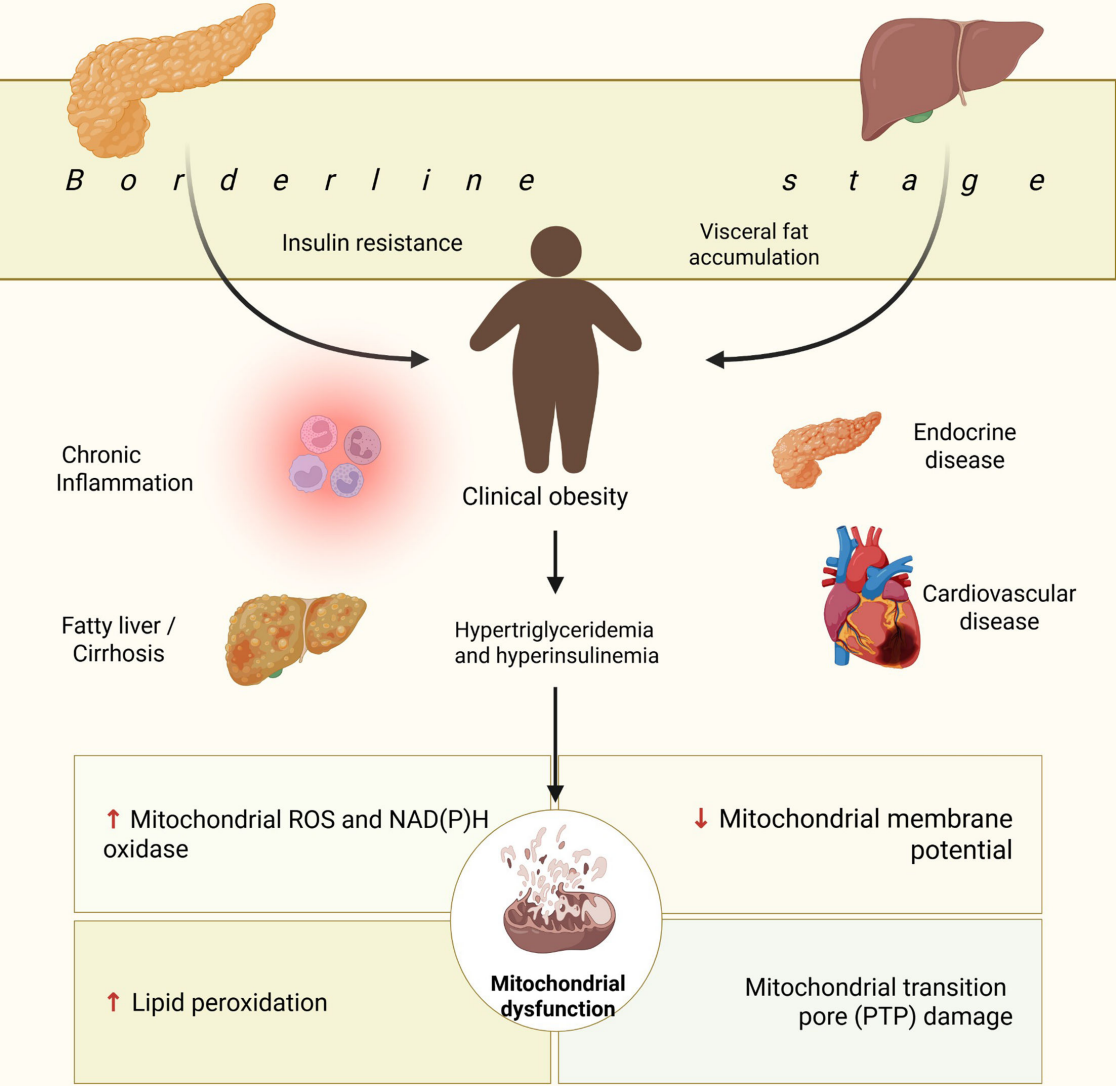
Summary of the borderline stage characterized by insulin resistance and visceral fat accumulation, and its implications in the development of chronic degenerative diseases.

Evidence suggests that mitochondrial dysfunction is an early contributor to metabolic impairment during the borderline stage of obesity. Impaired mitochondrial oxidative phosphorylation in obesity-prone individuals may lead to a metabolic shift favoring anaerobic glycolysis. This phenomenon reduces ATP production efficiency and promotes hepatic lipogenesis by diverting substrates toward lipid synthesis rather than complete oxidation. Reduced mitochondrial activity—particularly in liver and muscle tissues—has been linked to decreased expression or activity of key oxidative enzymes such as citrate synthase and cytochrome c oxidase, further exacerbating lipid accumulation and insulin resistance. These changes constitute an early bioenergetic failure that precedes overt metabolic dysfunction [104] (Figure 5).

In a murine model, a high-fat, high-sucrose diet (HFHSD) led to mitochondrial dysfunction in skeletal muscle, driven by excessive production of reactive oxygen species (ROS) triggered by hyperglycemia and hyperlipidemia. These alterations were attributed to mitochondrial hyperactivity and increased NAD(P)H oxidase activity in response to energy substrate overload [137]. These findings underscore the importance of considering mitochondrial function and individual susceptibility when developing therapeutic strategies for obesity and metabolic disorders.

Interindividual variability in response to high-fat diets may determine progression from preclinical to clinical obesity. In a study using a rodent model, rats were fed an HFHSD for eight weeks and then stratified based on weight gain. The upper third was classified as diet-induced obesity (DIO) animals, while the lower third was considered diet-resistant (DR). Compared with DR rats, DIO animals exhibited higher levels of brain lipid peroxidation, lower circulating ghrelin levels, increased mitochondrial ROS production, and reduced mitochondrial membrane potential—indicative of compromised mitochondrial integrity. Since membrane potential reflects the status of the mitochondrial permeability transition pore (PTP), these results suggest that early mitochondrial damage may play a pivotal role in the development of diet-induced obesity [138] (Figure 5).

Interestingly, the pharmacological agent metformin, widely used in the treatment of type 2 diabetes, exerts part of its therapeutic effect through mild inhibition of complex I of the mitochondrial electron transport chain. This inhibition reduces ATP production and increases AMP levels, thereby activating AMP-activated protein kinase (AMPK), which in turn suppresses hepatic gluconeogenesis and lipogenesis. This mechanism not only improves glycemic control but may also counteract some of the early mitochondrial and metabolic alterations observed in the borderline stage of obesity [139].

### Hypothalamic alterations and their impact on metabolic regulation

Moraes et al. demonstrated that high-fat diet (HFD) consumption induces neuronal apoptosis and synaptic loss in the arcuate nucleus and lateral hypothalamic areas of rodents. Notably, this effect was dependent on diet composition rather than caloric intake, as caloric restriction alone did not attenuate apoptotic markers. Toll-like receptor 4 (TLR4) was found to play a dual role—activating proinflammatory signaling pathways associated with leptin and insulin resistance, while also providing partial protection against further apoptotic damage [140].

Additional evidence supports the involvement of hypothalamic inflammation in diet-induced obesity. In a separate study, deletion of IKKβ in astrocytes was shown to prevent obesity and reduce hypothalamic inflammation. In this murine model, tamoxifen-induced deletion of IKKβ before HFD exposure did not prevent weight gain or glucose intolerance. However, when mice were first exposed to HFD for six weeks followed by tamoxifen-induced deletion, a significant recombination was observed in the mediobasal hypothalamus (MBH). This approach led to reduced weight gain, decreased food intake, and increased energy expenditure. Furthermore, IKKβ deletion improved insulin sensitivity and attenuated astrocyte activation and inflammatory signaling in the hypothalamus. These findings suggest that astrocytic IKKβ plays a crucial role in regulating hypothalamic inflammation and energy homeostasis in obesity [141]. Moreover, these alterations could occur from the borderline stage.

### Assessment of progression to clinical obesity

While the prediabetes model provides a helpful framework for identifying early disturbances in glucose metabolism, it does not fully capture the complexity of clinical obesity and its systemic implications. Metabolic homeostasis is often compromised before measurable increases in blood glucose levels occur, particularly in gradual decline in whole-body insulin sensitivity with stable β-cell function. Indeed, evidence indicates that in patients before they debut with T2DM, the alterations could occur 10 years earlier [142]. In contrast, the proposed borderline model provides a more integrative perspective, encompassing glucose dysregulation, alterations in lipid metabolism, inflammation, insulin resistance, and mitochondrial dysfunction.

Positioned within the spectrum of preclinical and clinical obesity, the borderline framework facilitates a broader understanding of early pathophysiological changes. As such, it may serve as a more effective foundation for developing preventive and therapeutic strategies to delay or halt the progression of chronic degenerative diseases.

## Therapeutic interventions

Clinical obesity significantly impairs quality of life due to its association with cardiovascular complications, metabolic syndrome, and T2DM. This section outlines therapeutic strategies for managing the clinical phase of obesity.

Lifestyle modification remains the cornerstone of initial management and should be prioritized as the first-line intervention. Pharmacological treatment should be individualized based on a comprehensive assessment of comorbidities (e.g., cardiovascular risk), potential adverse effects (particularly in older adults), treatment burden, and the patient’s goals and preferences [143].

### Insulin resistance approach

Metformin, an oral hypoglycemic agent, remains the first-line pharmacological therapy for T2DM and insulin resistance, either as monotherapy or in combination with other agents. Its primary mechanism of action involves the inhibition of hepatic gluconeogenesis, coupled with enhanced glucose uptake in skeletal muscle and improved peripheral insulin sensitivity. Metformin effectively lowers glycated hemoglobin (HbA1c) levels by approximately 1.5% to 2%. Although it does not exert a direct weight-reducing effect, its use has been associated with modest reductions in blood pressure, serum cholesterol, triglycerides, and biomarkers of vascular inflammation [144].

Recently, glucagon-like peptide-1 receptor agonists (GLP-1 RAs) have emerged as a cornerstone in the management of T2DM, particularly in patients with insulin resistance and high cardiovascular risk. GLP-1 is an incretin hormone secreted by intestinal L-cells in response to food intake, which stimulates glucose-dependent insulin secretion, suppresses glucagon release, and delays gastric emptying [145]. These physiological effects provided the basis for the development of GLP-1 analogs, including exenatide, liraglutide, and semaglutide, synthetic peptides with 53%, 97%, and 94% sequence homology to endogenous GLP-1, respectively. Unlike the native hormone, these compounds are resistant to degradation by dipeptidyl peptidase-4 (DPP-4), thereby prolonging their half-life and enhancing clinical efficacy [146].

Semaglutide is available in both oral and subcutaneous formulations, whereas other GLP-1 RAs, such as exenatide, liraglutide, lixisenatide, and dulaglutide, are administered exclusively via the subcutaneous route [145]. The most common adverse effects associated with this pharmacological class are gastrointestinal, primarily nausea and vomiting [147]. Nevertheless, GLP-1 RAs have demonstrated significant clinical efficacy, especially in individuals at high cardiovascular risk, due to their weight-reducing properties and beneficial effects on endothelial function, inflammation, oxidative stress, and myocardial protection [148].

Glucagon-like peptide-1 receptor agonists (GLP-1RAs) have shown robust efficacy in promoting clinically significant weight loss and improving metabolic parameters in individuals with obesity. While most evidence has focused on patients with insulin resistance or metabolic comorbidities, emerging data indicate that GLP-1RAs also confer benefits in individuals with obesity who do not exhibit overt metabolic disturbances. Beyond improving glycemic control, these agents reduce hepatic fat accumulation, enhance insulin sensitivity, and lower cardiovascular risk. Sustained weight reduction achieved with GLP-1RAs may therefore prevent progression to more severe obesity and mitigate long-term cardiometabolic complications. Consequently, therapeutic decisions should be individualized according to clinical characteristics, comorbidities, and socioeconomic context [149–151].

Randomized controlled trials provide robust evidence that GLP-1RAs, administered as monotherapy or in dual formulations, are effective for weight management in adults without diabetes. In this population, tirzepatide (dual GLP-1 and GIP receptor agonist) 15 mg once weekly achieved weight reductions of up to 17.8% (95% CI, 16.3–19.3) at 72 weeks, semaglutide 2.4 mg once weekly yielded reductions of up to 13.9% (95% CI, 11.0–16.7) at 68 weeks, and liraglutide 3 mg once daily produced reductions of up to 5.8% (95% CI, 3.6–8.0) at 26 weeks [152]. Although the overall safety profile is favorable, gastrointestinal adverse events—reported in more than 80% of participants—remain a significant limiting factor, and together with treatment costs, account for a substantial proportion of discontinuations [152]. Continuous clinical monitoring and gradual dose titration are therefore crucial for optimizing tolerability, promoting adherence, and maximizing the therapeutic benefits of GLP-1–based interventions [152,153].

Controlled studies have shown that, following treatment discontinuation, patients experience a significant regain of the weight lost during the first year. This ‘rebound’ phenomenon varies depending on the pharmacological agent but is generally considered clinically relevant. A recent meta-analysis reported an average weight gain of 9.7 kg after discontinuation of semaglutide or tirzepatide. In contrast, liraglutide was associated with a gain of 2.2 kg, regardless of treatment duration or nutritional counseling after withdrawal [154]. These findings underscore the need to consider pharmacological regimens as chronic interventions and to implement gradual tapering strategies combined with sustained caloric reduction, regular physical activity, and dietary restraint, in order to minimize weight regain and promote long-term weight loss maintenance. Early intervention with GLP-1RAs thus represents a promising strategy for comprehensive obesity management and prevention of obesity-related complications.

The latest guidelines from the American Diabetes Association (ADA) recommend GLP-1 RAs and SGLT2 inhibitors in the following scenarios [143]:

- High atherosclerotic cardiovascular disease (ASCVD) or heart failure risk: Prefer GLP-1 RAs or SGLT2 inhibitors.
- Chronic kidney disease (GFR 20–60 ml/min/1.73 m²): Use GLP-1 RAs or SGLT2 inhibitors, noting reduced glycemic benefit below 45 ml/min/1.73 m²
- Metabolic dysfunction-associated steatotic liver disease (MASLD): Consider GLP-1 RAs or dual GLP-1/GIP agonists
- Insulin-dependent therapy: Add or maintain GLP-1/GIP agents with appropriate insulin dose adjustments.

Thus, with appropriate intervention based on GLP-1RAs, therapeutic goals can be achieved, and therefore the characteristic conditions: hypertriglyceridemia, hyperinsulinemia, and low-grade inflammation markers.

### Hypertriglyceridemia management

A wide range of factors, including diet, genetic predisposition, stress, sleep patterns, medication or alcohol consumption, and sedentary behavior, modulate serum triglyceride levels. These determinants may promote hepatic triglyceride synthesis, inhibit peripheral lipolysis, or alter lipid clearance mechanisms [155]. Consequently, the clinical management of hypertriglyceridemia should include the identification and control of these factors to establish a comprehensive therapeutic strategy that combines dietary interventions, physical activity, and, when necessary, targeted pharmacotherapy.

In accordance with this, Broussard et al. demonstrated that a three-month intervention combining caloric restriction and physical exercise in individuals with obesity resulted in a significant reduction in serum triglyceride levels. Participants exhibited an approximate 10% decrease in body mass index, accompanied by a 57% improvement in insulin sensitivity, as assessed by the hyperinsulinemic-euglycemic clamp technique. This enhancement in insulin sensitivity was associated with a marked reduction in total serum triglyceride concentrations [156]. Moreover, individuals with higher baseline TAG levels showed a greater magnitude of reduction, suggesting that those with hypertriglyceridemia may derive more pronounced benefits from combined hypocaloric diet and regular exercise interventions [156]. Overall, these findings support the efficacy of lifestyle modifications as a first-line therapeutic strategy to improve lipid profiles and insulin sensitivity in patients with obesity and those situated in borderline stage. Pharmacological treatment should be reserved for moderate-to-severe hypertriglyceridemia, for which agents such as fibrates, niacin, and omega-3 fatty acids are recommended [112].

Severe hypertriglyceridemia represents a major metabolic risk factor associated with acute pancreatitis and atherosclerotic cardiovascular disease. In its pharmacological management, fibrate derivatives and omega-3 fatty acids (O3FA) are recommended as first-line therapies when serum triglyceride levels exceed 500 mg/dl, according to the AHA guidelines. In patients with mixed hyperlipidemia, statins may be used as adjunct therapy, as they have been shown to reduce serum triglyceride concentrations by 20% to 40% and to increase high-density lipoprotein cholesterol (HDL-C) levels by 5% to 10%. Moreover, statin therapy is associated with a significant reduction in the risk of atherosclerotic cardiovascular disease (ASCVD), establishing it as an essential component of the comprehensive management of dyslipidemia [157] Thus, depending on the magnitude of hypertriglyceridemia, the required treatments can be performed to prevent the transition to clinical obesity.

### Cardiovascular disease approach

The most recent guidelines from the European Society of Cardiology (ESC) emphasize the importance of reducing both fatal and non-fatal cardiovascular risk through the prevention and management of arterial hypertension [158]. Key recommendations include the following:

- Lifestyle modifications should be implemented for an initial period of three months. If the target blood pressure is not achieved, pharmacological treatment should be initiated.
- Secondary causes of hypertension should be investigated in individuals diagnosed before the age of 40, except young adults with obesity, in whom screening for obstructive sleep apnea is recommended as a first step.
- Self-monitoring of blood pressure is encouraged to promote patient empowerment and improve adherence to antihypertensive therapy.

In addition to blood pressure control, managing dyslipidemia is crucial for reducing cardiovascular risk. The joint guidelines of the ESC and the European Atherosclerosis Society (EAS) recommend statins as first-line therapy due to their substantial efficacy in lowering low-density lipoprotein cholesterol (LDL-C). Statins can reduce LDL-C levels by 20–55%, depending on the agent and dosage [159]. Furthermore, a meta-analysis of 28 randomized controlled trials demonstrated a 22% reduction in cardiovascular events for every 39 mg/dl (1 mmol/l) decrease in LDL-C [160].

### Preventive treatment in clinical obesity

Prevention of clinical obesity encompasses not only dietary quality and quantity but also the promotion of healthy sleep patterns and regular physical activity. Before the onset of obesity-related complications, there is often a variable preclinical period lasting several years. During this window, screening tests may detect individuals with borderline values, providing a critical opportunity for intervention to prevent disease progression.

The ADA recommends routine screening for diabetes and insulin resistance in all adults aged 45 years or older every three years, and annually in individuals with a BMI of 25 kg/m² or higher who present one or more additional risk factors. These include physical inactivity, hypertension, dyslipidemia, family history of diabetes, history of gestational diabetes, previous diagnosis of prediabetes (HbA1c ≥ 5.7%), polycystic ovary syndrome, and specific racial or ethnic backgrounds associated with increased risk [161].

The implementation of a screening program to identify individuals in a borderline stage of obesity would entail additional costs associated with laboratory tests (e.g. lipid profile, HOMA-IR, inflammatory markers) and more frequent clinical evaluations, potentially increasing the demand for diagnostic resources and imposing a burden on healthcare systems. Nevertheless, evidence suggests that the potential benefits substantially outweigh these limitations. In the United States, obesity has been estimated to increase medical expenditures by approximately US $1,861 per person per year, corresponding to an additional US $172.74 billion in direct medical costs nationally [162,163]. Previous analyses similarly reported an economic burden exceeding US $260 billion in 2016 [164], and approximately US $147 billion as early as 2008 [165]. These findings highlight that the management of advanced metabolic and cardiovascular complications entails considerably higher expenditures compared with the implementation of preventive strategies. Consequently, recognizing an intermediate stage such as borderline obesity would enable earlier interventions—such as intensive lifestyle modification and, in selected cases, pharmacological therapy—aimed at preventing progression to clinical obesity and reducing long-term healthcare costs associated with T2DM, hypertension, dyslipidemia, and cardiovascular disease [166].

## General analysis

Based on current evidence, the conceptual application of the term borderline within the framework of preclinical and clinical obesity appears both feasible and clinically relevant. This perspective allows for a more comprehensive understanding of obesity-related pathophysiology by shifting the focus beyond glycemic control to encompass broader metabolic disturbances—particularly those related to lipid metabolism, inflammation, and insulin signaling.

The relationship between fatty acids, triglycerides, and insulin receptor signaling is complex and influenced by various individual factors, including diet, age, genetic predisposition, and physical activity. These elements influence cellular metabolism, altering the concentrations and profiles of circulating lipids, glucose and insulin levels, inflammatory mediators, and markers of oxidative and endoplasmic reticulum stress. Collectively, these changes impair glucose transport, promote lipid accumulation, reduce insulin sensitivity, and increase the risk of developing T2DM, metabolic syndrome, and cardiovascular disease.

As discussed throughout this review, borderline parameters represent a transitional stage in which early metabolic dysfunction is already present but has not yet crossed clinical diagnostic thresholds. This stage should not be interpreted as metabolically neutral; instead, it may represent a critical inflection point. Early identification and intervention at this stage could help prevent or delay the progression to full-blown disease. For instance, detecting HOMA-IR values between 2.0 and 2.5, triglycerides between 150 and 200 mg/dl, or CRP levels above 0.8 mg/l may allow clinicians to initiate timely lifestyle or pharmacological strategies. They should be viewed as metabolic warning signs that warrant closer monitoring and action—not passive findings. At the population level, recognizing this transitional stage could also improve screening practices and reduce long-term healthcare costs before irreversible complications develop.

Although we acknowledge the limitations inherent to observational data, we would like to underscore that several large-scale, population-based studies utilizing NHANES cohorts have identified clear associations between early metabolic alterations, particularly hyperinsulinemia, hypertriglyceridemia, and excess adiposity, and the subsequent development of T2DM, NAFLD, and cardiometabolic multimorbidity.

In a retrospective analysis of NHANES and NHANES III data, Stokes et al. [167] demonstrated that individuals who maintained obesity or gained weight between early adulthood and midlife had a three-fold increased risk of developing T2DM compared with those who remained non-obese throughout adulthood. This study highlights the predictive value of long-term weight trajectories and underscores the preventive potential of identifying individuals at risk early in the metabolic continuum. Similarly, Cameron et al. [168] quantified the population-level burden of obesity on incident diabetes using data from NHANES (2001–2016) and MESA. Authors reported that obesity accounted for 30–53% of new diabetes cases, with variability across sex and ethnic groups. These findings support the public health relevance of stratifying individuals in transitional stages, even before a clinical diagnosis is made.

Moreover, Wang et al. (2023) [169] found that adults in NHANES III who experienced obesity earlier in life had a significantly higher prevalence of NAFLD compared with those who became obese later or remained non-obese. Evidence suggests that early adiposity, which likely overlaps with the proposed borderline stage, can have lasting impacts on hepatic health. Additionally, a 2024 analysis by Gurevitz et al. [170] using NHANES 1999–2020 data showed that hypertriglyceridemia was strongly associated with multiorgan metabolic disease, including central obesity, NAFLD, and diabetes—even in the absence of full-blown clinical diagnoses. These biochemical indicators align closely with the metabolic profile we propose for the borderline stage. The evidence supports the clinical utility of defining a borderline stage of obesity based on biochemical and anthropometric markers. While further prospective and interventional studies are needed to validate this concept, the cited evidence strengthens the rationale for early risk stratification and personalized intervention before overt disease onset.

As previously proposed by our group, the *Exosomal Pentad* model describes the reciprocal communication among five metabolically active tissues—adipose, skeletal muscle, liver, pancreas, and intestine—mediated by extracellular vesicles. This model provides a theoretical scaffold to understand the systemic nature of obesity-related dysfunction and supports the notion that preclinical or borderline stages may be orchestrated through early alterations in exosomal signaling. Understanding how these vesicles propagate metabolic stress signals could lead to the identification of early biomarkers and therapeutic targets for metabolic disease prevention [102].

Ultimately, this conceptual framework presents significant opportunities for future research. Standardizing diagnostic criteria for the borderline stage, exploring its progression through longitudinal studies, and developing targeted early interventions are all key priorities for addressing this condition (Figure 5). As research advances, this approach may support the design of personalized strategies that complement current therapies for obesity, contributing to a more preventive and anticipatory model of care.

## Open Access

This article has been published open access under our Subscribe to Open programme, made possible through the support of our subscribing institutions, learn more here: https://portlandpress.com/pages/open_access_options_and_prices#conditional

## Abbreviations

BMI: body mass index
GWAS: genome-wide association studies
HPA axis: hypothalamic–pituitary–adrenal axis
PRS: polygenic risk scores
T2DM: type 2 diabetes mellitus
